# Constrained-Spherical Deconvolution Tractography in the Evaluation of the Corticospinal Tract in Glioma Surgery

**DOI:** 10.3389/fsurg.2021.646465

**Published:** 2021-07-29

**Authors:** Zhiyuan Sheng, Jinliang Yu, Zhongcan Chen, Yong Sun, Xingyao Bu, Meiyun Wang, Can Sarica, Juha Hernesniemi, Bradley J. Nelson, Ajmal Zemmar, Josue M. Avecillas-Chasin

**Affiliations:** ^1^Juha Hernesniemi International Neurosurgery Center, Zhengzhou University People's Hospital (Henan Provincial People's Hospital), Zhengzhou, China; ^2^Department of Radiology, People's Hospital of Zhengzhou University (Henan Provincial People's Hospital), Zhengzhou, China; ^3^Division of Neurosurgery, Department of Surgery, University of Toronto, Toronto, ON, Canada; ^4^Multi Scale Robotics Laboratory, ETH Zurich, Zurich, Switzerland; ^5^Department of Neurosurgery, Center for Neuromodulation, Mount Sinai Health System, New York, NY, United States

**Keywords:** glioma, tractography, white matter, surgical resection, corticospinal tract

## Abstract

**Introduction:** Tractography has demonstrated utility for surgical resection in the setting of primary brain tumors involving eloquent white matter (WM) pathways.

**Methods:** Twelve patients with glioma in or near eloquent motor areas were analyzed. The motor status was recorded before and after surgery. Two different tractography approaches were used to generate the motor corticospinal tract (CST): Constrained spherical deconvolution probabilistic tractography (CSD-Prob) and single tensor deterministic tractography (Tens-DET). To define the degree of disruption of the CST after surgical resection of the tumor, we calculated the percentage of the CST affected by surgical resection, which was then correlated with the postoperative motor status. Moreover, the fractional anisotropy (FA), mean diffusivity (MD), axial diffusivity (AD), and radial diffusivity (RD) of the CST generated by the CSD-Prob and the Tens-DET was measured and compared between the ipsilesional and contralesional side.

**Results:** The CST was identified in all patients and its trajectory was displaced by the tumor. Only the CSD-Prob approach showed the CST with the characteristic fan-like projections from the precentral gyrus to the brainstem. Disruption of the CST was identified in 6/6 with postoperative motor deficit by CSD-Prob approach and in 5/6 in the Tens-DET. The degree of disruption was significantly associated with the motor deficit with the CSD-Prob approach (*rho* = −0.88, *p* = 0.021). However, with the Tens-DET approach the CST disruption did not show significant association with the motor function (*rho* = −0.27, *p* = 0.6). There was a significant decrease in FA (*p* = 0.006) and a significant increase in MD (*p* = 0.0004) and RD (*p* = 0.005) on the ipsilesional CST compared with the contralesional CST only with the CSD-Prob approach.

**Conclusion:** CSD-Prob accurately represented the known anatomy of the CST and provided a meaningful estimate of microstructural changes of the CST affected by the tumor and its macrostructural damage after surgery. Newer surgical planning stations should include advanced models and algorithms of tractography in order to obtain more meaningful reconstructions of the WM pathways during glioma surgery.

## Introduction

Surgical resection is part of the multidisciplinary treatment of gliomas and the main goal is to achieve a safe gross total resection (1). When these tumors are located near or within eloquent brain regions, the surgical resection poses significant risk of damaging those regions. In these cases, mapping the eloquent white matter (WM) pathways related with the tumor has been considered useful to plan the surgical approaches (2). Diffusion-weighted image (DWI) is a magnetic resonance image (MRI) sequence that is based on the movement of the water molecules in every voxel of brain tissue. In the white matter, due to the increased hindrance to diffusion across the axonal membranes, the movement of the water molecules detected by DWI follow the directions of the major axonal bundles. Tractography is a technique that models the white matter pathways in the brain using DWI data. Tractography has demonstrated to be useful for surgical resection in the setting of primary brain tumors in eloquent areas, as long as the WM pathways generated are anatomically accurate (3).

The single-tensor model is the most commonly used to estimate WM fiber orientations from DWI data. This model defines a single predominant orientation of the movement of water molecules in each voxel. Likewise, the deterministic algorithm is the tracking method based on the principal diffusion direction modeled voxel by voxel (4–6). The combination of the single-tensor model with the deterministic algorithm is one of the first techniques used for tractography and is currently the most commonly used in surgical planning stations. The effect of intra-axial tumors on the WM (edema, infiltration, displacement, etc.) may affect the ability of less contemporary tractography techniques such as single-tensor deterministic to provide accurate reconstructions of the WM pathways (7). Ideally, the streamlines generated by tractography, which is the 3D representation of the tract of interest, should accurately define the trajectory and roughly approximate the cross-sectional area of the real underlying WM pathway. This way, a reliable plan for safe resection can be tailored based on the relationship between the tumor and the WM pathways.

Meanwhile, high-order tractography methods have been developed in the past 20 years. These approaches have demonstrated to improve the anatomical accuracy and the validity of the results (8). Constrained-spherical deconvolution (CSD) tractography has been previously evaluated in patients with intra-axial brain tumors. This approach provided valuable quantitative and qualitative information that has not been obtained with single-tensor approaches to aid *preoperative* planning for these patients (9). When comparing deterministic and probabilistic tractography algorithms, high-order tractography (q-ball probabilistic) techniques demonstrated better sensitivity and specificity than tensor-based approaches to locate the functional motor pathways using intraoperative stimulation as the gold standard (10). These data also evidenced the very poor sensitivity of the current tensor-based approaches in finding the ventral aspects of the motor corticospinal tract. The effect of edema and tumor mass effect further reduced the sensitivity of these approaches.

Considering that the aim of tractography for brain tumor surgery is to obtain meaningful reconstructions of the major WM pathways, there is still controversy on the advantage of advanced approaches in terms of its utility in clinical practice. Clinically meaningful reconstructions are urgently needed as image-guided minimally invasive approaches (laser ablation, focused ultrasound, radiosurgery, etc.) are becoming more popular in the management of tumors in eloquent areas or deeper locations (11, 12). The aim of this study is to investigate the utility of high-order tractography approaches in patients with intra-axial tumors and their clinical outcomes. To this aim, we analyzed patients operated for gliomas near or within motor areas. We compared the single-tensor deterministic approach (Tens-DET), which is the available approach in surgical planning stations with the CSD probabilistic approach (CSD-Prob) as a high-order more advanced tractography approach.

## Methods

### Patient Selection

Fifty-three patients underwent glioma resection surgery with subsequent pathological confirmation according to the 2016 WHO brain tumor classification criteria (13) at the Henan Provincial People's Hospital between January 2017 and January 2019. From these 53 patients, we selected patients based on the following criteria: tumor resection surgery, tumors within or near motor eloquent areas, and mild motor deficit (4/5) at the clinical presentation that would suggest displacement rather than damage of the motor corticospinal tract (CST). Additionally, we excluded patients if they had significant postoperative brain shift or edema that would preclude accurate co-registration of the preoperative and postoperative acquisitions. We recorded the clinical and demographic data, extent of resection, and permanent postoperative motor deficits (no recovery after last follow-up ~2 months after surgery). The motor deficit was evaluated with the medical research council scale ranging from 1 (no contraction) to 5 (normal muscle strength). Gross total resection was defined as resection of more than 90% of the tumor volume. The local ethics board approved the study and consent was provided by all patients.

### Data Acquisition, Pre-processing, and Registration

The MRI data were acquired using a 3.0T whole-body MRI scanner (Siemens electronic medical system). The 3-dimensional preoperative and postoperative (within 2 days after surgery) T1-weighted images with gadolinium were acquired with the following imaging parameters: repetition time = 2,300 ms, echo time = 2.3 ms, inversion time = 900 ms, slice thickness = 1 mm, flip angle = 8, number of excitations = 1, field of view = 240 * 240 mm^2^, voxel sizes = 0.9 * 0.9 * 1.0 mm^3^, and acquisition time = 421 s. Preoperative high-angular resolution diffusion images (HARDI) were acquired with a thickness of 4.0 mm that covers the whole brain with the following parameters: repetition time = 3,000 ms, echo time = 69 ms, flip angle = 180, b-values = 1,000 s/m^2^, field of view = 220 * 220 mm^2^, voxel sizes = 1.7 * 1.7 * 4.0 mm^3^, number of excitations = 4, contiguous slices = 25, acquisition time = 257 s, and 64 gradient directions. The data was pre-processed using MRtrix and FSL tools (14, 15). Eddy-current, bias-field, and subject movement were corrected (16). The response function and the spherical deconvolution were estimated, and fiber orientation distributions (FOD) were obtained. Finally, linear registration was utilized to co-register the preoperative (T1-weighted and diffusion images) with the postoperative acquisitions. Using the postoperative acquisitions, we contoured the resection cavity, and this volume was later used to calculate the disruption of the CST by the surgical resection.

### Tractography Analysis

**Figure 1:**
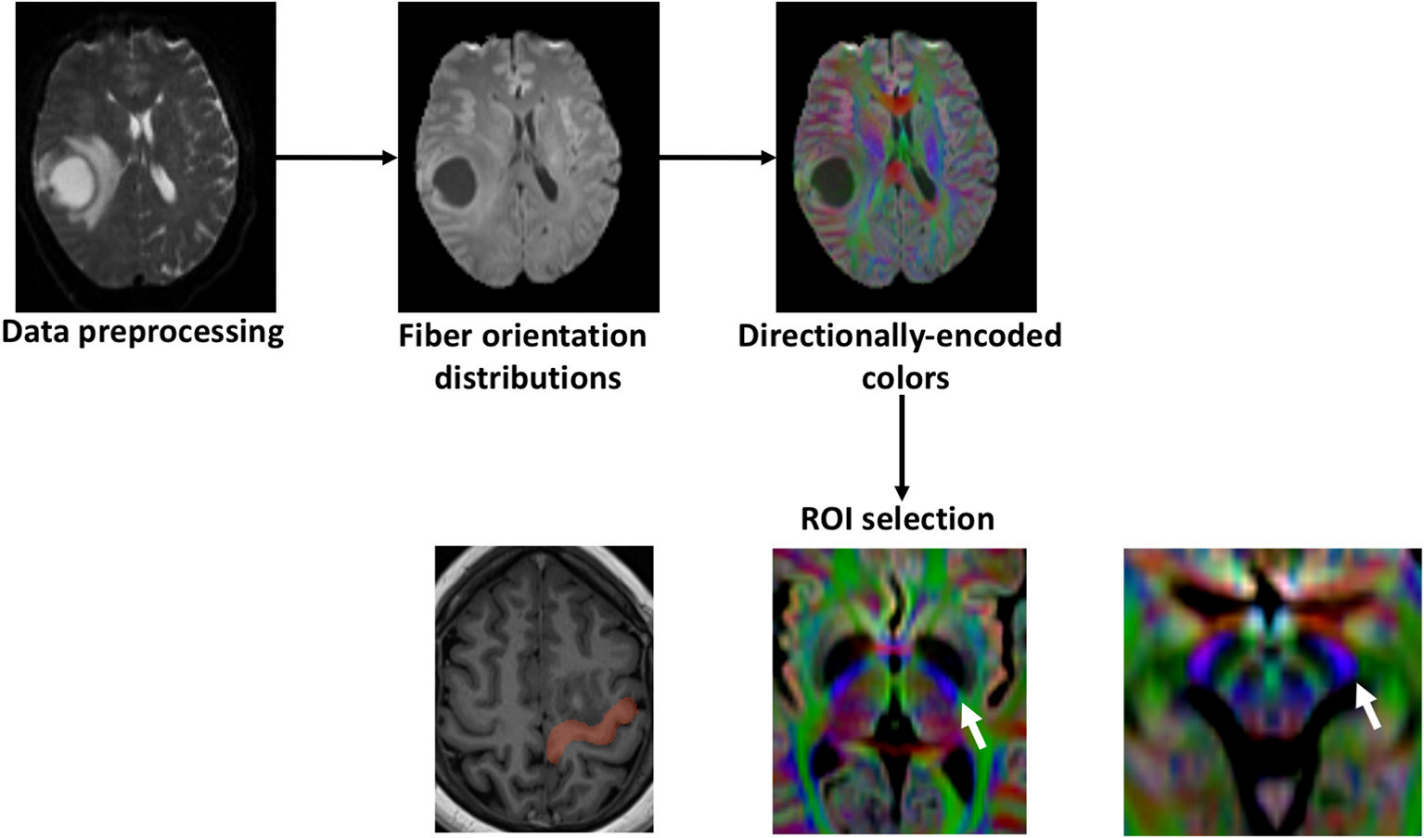
Study outline. First, diffusion weighted images were preprocessed with subject-motion, Eddy current distortion, and bias field correction. Fiber orientation distributions were calculated with constrained-spherical deconvolution and the directionally-encoded colors were obtained. The precentral gyrus was delineated using the T1 and defined as the seed region of interest (ROI). The other inclusion ROIs were selected in the directionally-encoded colors sequence based on the described trajectory of the motor CST. The posterior half of the posterior limb of the internal capsule and the posterolateral third of the cerebral peduncle (white arrows).

The CST was generated using two different approaches: the CSD-probabilistic tractography (CSD-Prob) and the single tensor deterministic tractography (Tens-DET). We defined three regions of interest (ROIs) based on the neuroanatomy of the CST as follows (17): Seed ROI: The precentral gyrus. Inclusion ROIs (10 mm): one sphere at the posterior half of the posterior limb of the internal capsule and one at the posterolateral third of the cerebral peduncle (Figure 1). The fiber tracking was performed in the ipsilesional and contralesional side with 10,000 streamlines, cut-off: 0.15 (FOD amplitude for CSD-Prob and FA for Tens-DET), step size: 0.5 × voxelsize, angle threshold: 45, and seeding density 10^3^ × the number of selected streamlines. To determine the microstructural changes of the CST as a result of the proximity of the tumor, we calculated a series of diffusion scalars including fractional anisotropy (FA), mean diffusivity (MD), axial diffusivity (AD), and radial diffusivity (RD) along the ipsilesional and contralesional CST. To this aim, we used the map of the motor CST to sample the DTI scalars along the CST generated by the Tens-DET and the CSD-Prob. These parameters are indirect measures of white matter structural integrity (14). Finally, the degree of surgical damage of the CST, henceforth referred to as “tract disruption,” was defined as follows: We first generated the ipsilesional corticospinal tract (CST) of each patient with a fixed arbitrary number of streamlines (10,000), which are the 3D representation of the trajectory of a given WM bundle (5). We used the postoperative images to segment the tumor resection cavity. Then, we subtracted the CST streamlines passing through the cavity of the tumor using MRtrix tools. We then defined the disruption of the CST as the relative percentage of streamlines excluded by the resection cavity. Finally, we used this percentage to correlate with the motor status.

### Statistical Analyses

The diffusion scalars calculated from the CST (FA, MD, RD, and AD) are values between 0 to 1 and were compared between the ipsilesional and contralesional side using the Wilcoxon test. The correlation analysis of the CST disruption with the postoperative motor status was performed using the Spearman correlation test. We used the SPSS software package 22.0 (IBM Corp, Armonk, NY) and the significance level was set at *p* < 0.05.

## Results

**Figure 2:**
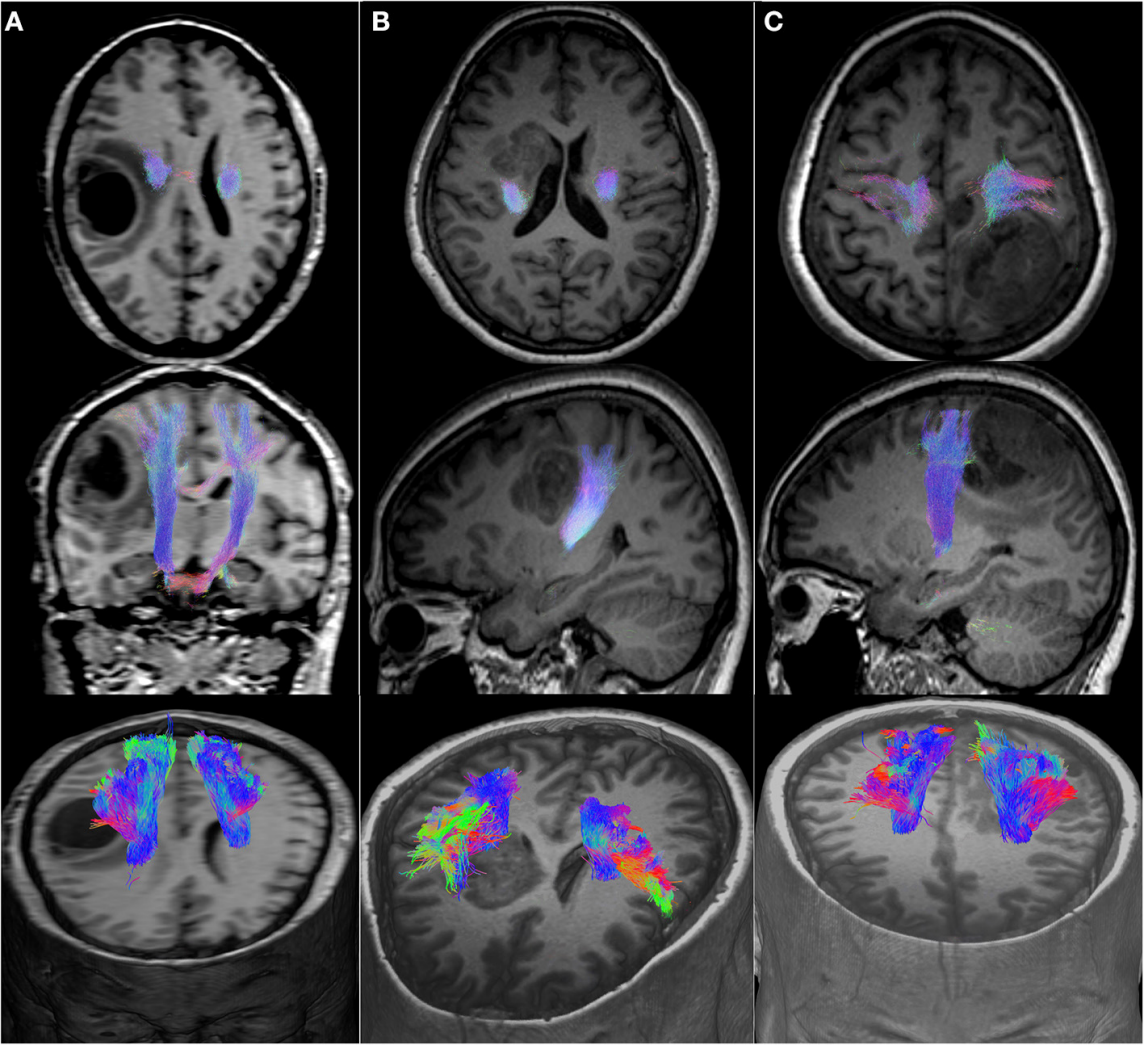
Motor CST displacement. **(A)**, Axial, coronal, and 3D view of a medial displacement of the CST. **(B)**, Axial, sagittal, and 3D view of a posterior displacement of the CST. **(C)**, Axial, sagittal, and 3D view of an anterior displacement of the CST.

**Figure 3:**
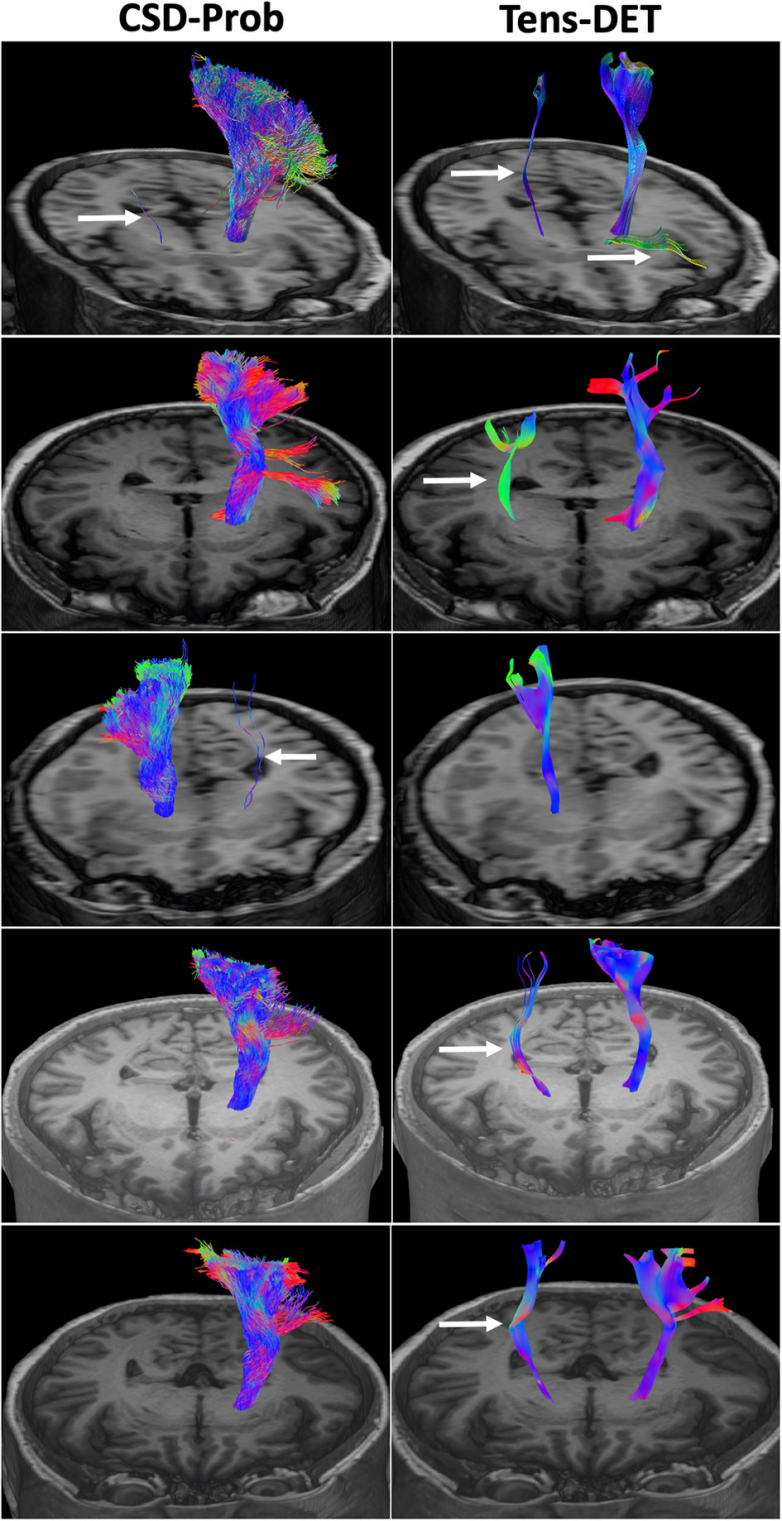
Comparison of the CSD-Prob and Tens-DET approaches in 5 subjects. The left panels show the 3D reconstruction of the CST by the CSD-Prob with the fan-like configuration of the cortical projections of the CST. The right panels show the CST by the Tens-DET approach with a straight trajectory to the dorsal part of the precentral gyrus, absence of the ventral part of the motor CST, and more prevalence of spurious fibers than the CSD-Prob (arrows).

**Figure 4:**
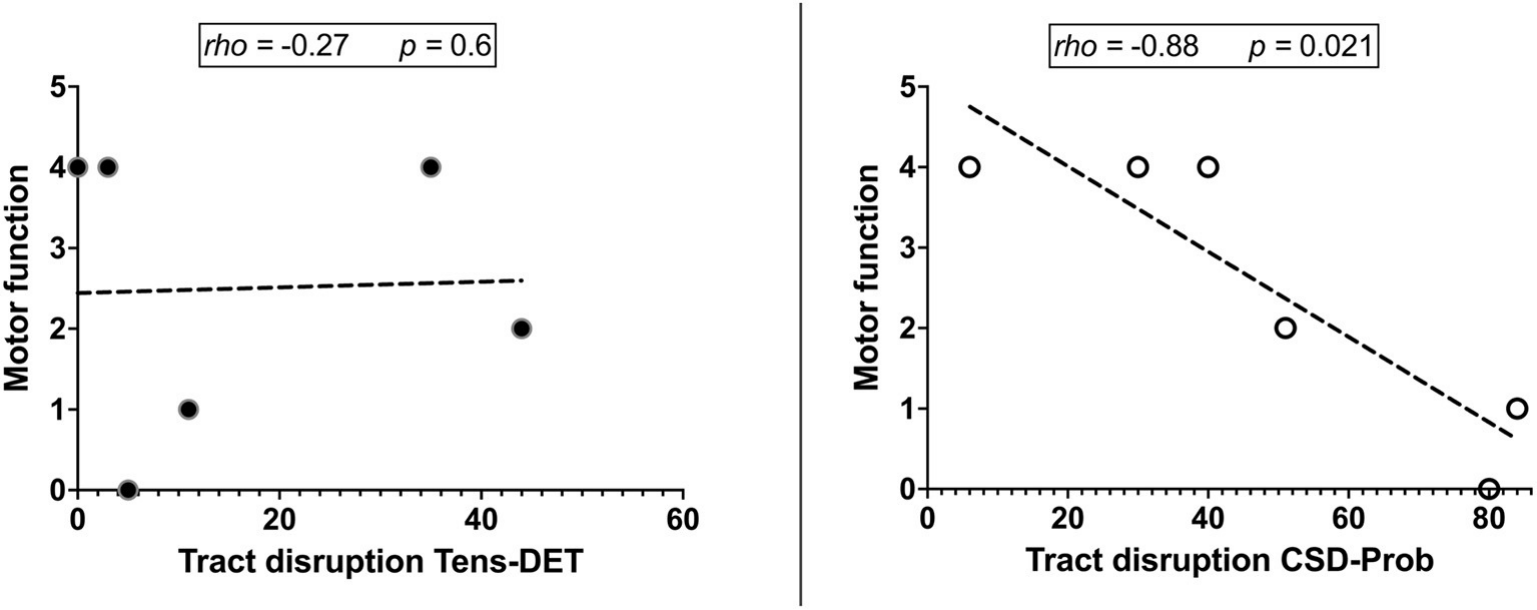
Correlation analyses show that the degree of motor deficit had a strong negative correlation with the CST disruption by the tumor resection. This significant association was found with the CSD-Prob approach and not with the Tens-DET approach likely due to the missing parts of the ventral part of the precentral gyrus.

**Figure 5:**
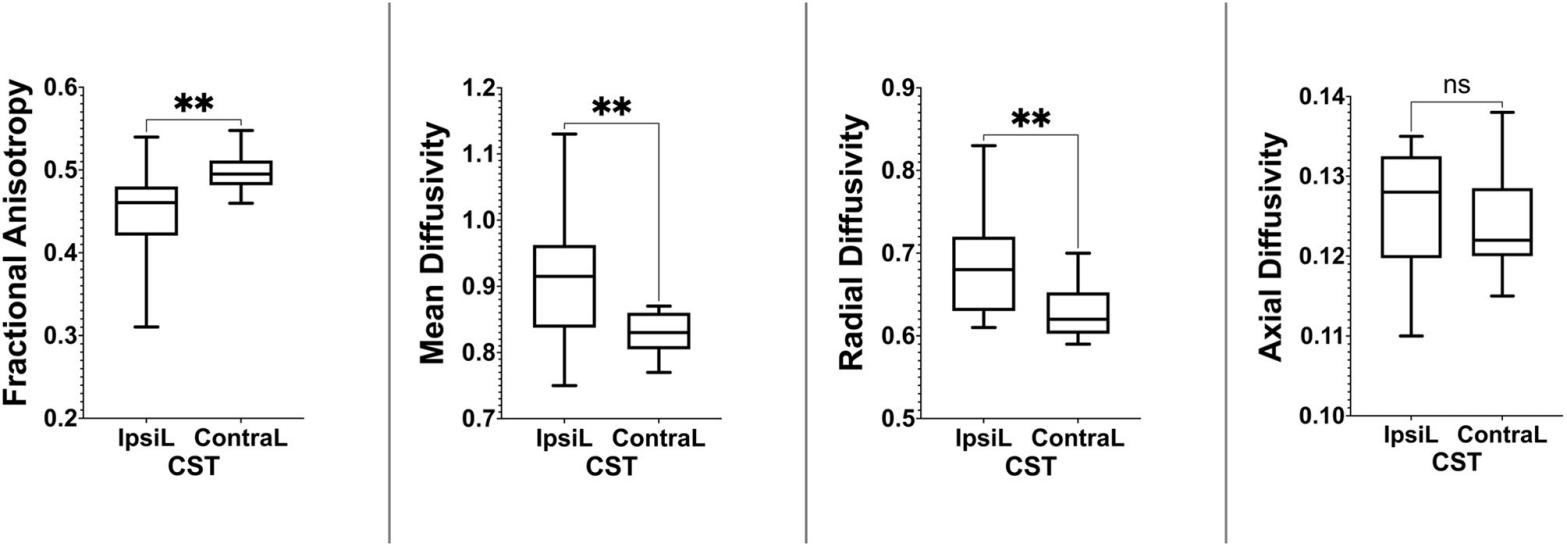
Boxplots showing statistically significant differences in fractional anisotropy, mean diffusivity, and radial diffusivity between the ipsilesional and contralesional side only with the CSD-Prob approach. **P ≤ 0.01.

We included 12 patients, six men and eight women with a mean age of 50 years (detailed clinical and demographic data are shown in Table 1). Pathological results yielded WHO grade II glioma in four patients and WHO IV glioblastoma (GBM) in eight patients. Preoperatively, six patients were neurologically intact and six presented with mild motor deficit (grade 4/5). Gross total resection was achieved in 9 patients. The CST was identified in all patients with the two tractography approaches with different patterns of displacement (Figure 2). The CSD-Prob approach showed the CST with the characteristic fan-like configuration from the dorsal and ventral precentral gyrus to the brainstem. The Tens-DET approach showed a straight trajectory from the dorsal precentral gyrus to the brainstem missing the ventral part of the precentral gyrus. This approach also underestimated the width of the CST and showed more spurious streamlines as compared with the CSD-Prob approach (Figure 3). Among the six patients with postoperative motor deficit, the CSD-Prob approach revealed CST disruption in different degree, and this was strongly associated with the degree of motor deficit in these patients (*rho* = −0.88, *p* = 0.021). The Tens-DET approach on the other hand, showed disruption of the CST in five of the six patients with motor deficit without significant association in the motor status (*rho* = −0.27, *p* = 0.6) (Table 1, Figure 4). Finally, only the CSD-Prob identified a significant decrease in fractional anisotropy (*p* = 0.0006) and an increase in mean and radial diffusivity (*p* = 0.004, 0.005) of the CST between the ipsilesional and the contralesional hemisphere (Figure 5). There was no significant difference in the axial diffusivity with the CSD-Prob approach and with none of the diffusion scalars with the Tens-DET approach.

**Table 1:** Clinical and demographics data of the patients.

**Cases**	**Age/Gender**	**Diagnosis**	**Preop motor status**	**Postop motor status**	**CST disruption CSD-Prob (%)**	**Tens-DET (%)**
1	58/F	Glioblastoma	5/5 right	=	–	–
2	54/M	Glioblastoma	5/5 left	=	–	–
3	37/F	Astrocytoma II	5/5 right	2/5 right lower limb	51	44
4	51/F	Astrocytoma II	4/5 right	5/5 right	–	–
5	51/F	Glioblastoma	5/5 left	=	–	
6	28/M	Oligodendroglioma II	5/5 right	=	–	–
7	49/M	Glioblastoma	5/5 left	=	–	–
8	58/F	Glioblastoma	4/5 left	1/5 left	84	11
9	55/F	Glioblastoma	4/5 right	0/5 right	80	5
10	43/F	Astrocytoma II	4/5 left	4/5 left upper limb	6	0
11	60/M	Glioblastoma	4/5 right	1/5 right upper limb, 3/5 right lower limb	30	3
12	59/F	Glioblastoma	4/5 left	1/5 left upper limb, 2/5 left lower limb	40	35

*F, female; M, male; Preop, preoperative; Postop, postoperative. The motor status was defined by the medical research council scale ranging from 1 (no contraction) to 5 (normal muscle strength). Corticospinal tract (CST) disruption is the percentage of the streamlines affected by the surgical resection cavity. CSD-Prob, constrained-spherical deconvolution probabilistic tractography; Tens-DET, Tensor deterministic tractography*.

## Discussion

The CST was generated by two different tractography techniques in the setting of brain tumor and peritumoral edema. The CSD-Prob approach has demonstrated accuracy in the representation of the CST as previously demonstrated (9). The CSD-Prob also showed a meaningful reconstruction of the CST since there was a consistent degree of CST disruption significantly correlated with postoperative motor deficit. The Tens-DET approach showed limited accuracy to generate the CST with more spurious streamlines than the CSD-Prob approach. Most of these spurious streamlines originated at the level of the brainstem and continued propagating to the contralateral side following different bundles including contralateral CST, cerebellum, and optic radiations. This may be related with a more prevalence of crossing fibers in the brainstem and the difficulty of the Tens-DET to resolve crossing fibers. Modifications in the tracking parameters of the Tens-DET approach could have minimized the presence of these spurious streamlines but the comparison of different tracking parameters in the Tens-DET approaches is beyond the scope of this article. In three of the six cases with postoperative motor deficit, the disruption of the CST defined by the Tens-DET approach was not consistent with the degree of motor deficit, likely due to the missing parts of the ventral precentral gyrus. Also, only the CSD-Prob approach identified a significant decrease in FA and a significant increase in the MD and RD of the CST on the ipsilesional side. The changes in FA, MD, and RD may be interpreted as an alteration of the microstructure in the white matter close to the tumor due to displacement of the axons or increase in free tissue water. These changes are related with tumor infiltration or the effect of the peritumoral edema on the WM tracts (9).

Surgery for gliomas in eloquent areas can be challenging when the extent of resection is compromised by brain function. Patients with mild motor deficit, who usually respond to steroid treatment, may have WM pathways intact and the precise location of these pathways is desirable in order to protect them during surgery. In addition to special techniques such as awake craniotomy (18) or neurophysiological monitorization, the use of tractography is a critical tool for the surgeon to minimize the risk of postoperative deficits during glioma surgery (2, 11, 12). The single-tensor deterministic technique, which is currently the most frequently used algorithm in surgical planning stations, prevents accurate preoperative identification of WM pathways. For example, Kinoshita et al., showed that the tractography reconstruction of the pyramidal tract was not accurate to estimate the cross-sectional area of the pathway despite being “anatomically” correct (19). Other authors on the other hand have found that tractography can accurately predict the position of the WM pathways and correlate with clinical outcomes (20, 21). These discrepancies are associated with major differences in algorithms, tracking settings, software, and data acquisition. For a WM pathway to be meaningful for surgical planning, it should represent the real anatomical trajectory and should closely estimate the cross-sectional diameter. This information would help, not only to understand the relationship between the tumor and the pathway, but also in the trade-off between extent of resection and the risk of postoperative deficit.

Reconstruction of WM pathways using tractography has several considerations to take into account. Usually, the ROIs for fiber tracking are identified based on neuroanatomy. The more ROIs, the more anatomical accuracy is achieved. Because of the distortion of the normal anatomy produced by the tumor, the use of directionally-encoded color (DEC) images would aid in the placement of the ROIs (22, 23). This image provides directional information of the white matter pathways: blue (superior-inferior direction), red (left-right direction), and green (anterior-posterior direction). By placing ROIs in blue areas of the theoretical location of the CST, it is possible to trace the trajectory of the CST by coupling anatomical and directional information. During tracking, single-tensor deterministic approaches use the main fiber orientation in the voxels to generate the pathway and this limits the anatomical accuracy because of the high prevalence of crossing fibers in the WM (24). Another problem of the single-tensor techniques is the inclusion of a number of false positive streamlines depending on the FA threshold established in the software. The present work and others have demonstrated that the fractional anisotropy is affected in the WM close to the tumor because of infiltration or edema. Therefore, false negatives will also limit the results of single-tensor tractography approaches.

CSD probabilistic tractography approaches are not free of limitations, as the sensitivity increases, the specificity of the WM pathway decreases as a substantial amount of false positive streamlines start to appear in the tracking process (25, 26). Current evidence has demonstrated that high-order tractography techniques may improve the false negative rate but have the problem of more false positive streamlines (25). However, the use of anatomical landmarks as waypoints, increases the accuracy in the tractography reconstruction (27, 28). Furthermore, when using probabilistic algorithms the use of thresholding would also improve the accuracy of the results (26). In this work, we used three ROIs using the directional information provided by the directionally-encoded colors sequence. This may help to minimize biases, which would be required to obtain meaningful reconstructions for surgical planning. Despite using clinical acquisitions, our results demonstrated that the CSD-Prob tractography reconstructions closely resemble the known anatomy of the motor CST (8, 29) in the presence of significant edema or displacement of the tracts. The disruption of the CST also showed strong correlation with postoperative motor outcomes. Due to the low sample size and other limitations, our results have to be interpreted with caution. However, we anticipate that the inclusion of high-order tractography approaches in newer surgical planning stations will allow to better characterize its utility for minimally invasive surgical techniques, including laser ablation (30), focused ultrasound, and radiosurgery.

The main limitations of our work include the small sample size and the retrospective nature of this analysis, which prevents a more comprehensive comparison of all tractography techniques available. The aim of this study was to demonstrate that the current methods of tractography used in surgical planning stations are not as reliable as other high-order tractography techniques available. Another limitation is the use of clinical acquisition instead of research acquisitions. Clinical acquisitions are usually limited by timing and patient comfort and therefore subsequent limitations in the acquisition parameters. Our DWI acquisition included highly anisotropic voxels and evidence suggest that this voxel reconstruction could add bias to the tractography reconstructions (31). Also, the diffusion scalars, particularly FA in regions containing crossing fibers, could be underestimated with acquisitions using anisotropic voxels (32). Therefore, it is recommended to use voxels with isotropic resolution in DWI acquisitions to improve the tractography results (33). Another consideration in the use of tractography for surgical planning is the practical meaning of the streamline reconstructions. The absolute number of streamlines lack biological meaning since the streamlines only represent a 3D model of the pathway. Also, the use of the streamlines as a measure of pathway density may be biased by the number of false positive connections (34). In this work, we used the percentage of streamline reduction as a semi-quantitative measure of tract disruption. Some evidence showed that the density of streamlines could be considered a marker of the structural integrity of the underlying WM pathway (35, 36). Streamline tractography has also been used to validate functional connectivity and to explain biological processes such as aging (37, 38).

## Conclusion

CSD-Prob accurately represented the known anatomy of the motor CST and provided a meaningful estimate of microstructural changes of the CST affected by the tumor and its macrostructural damage after surgery. Compared with CSD probabilistic approaches, single-tensor deterministic approaches seemed to underestimate the cross-sectional area of the CST in regions close to the tumor. Newer surgical planning stations should include advance tractography approaches in order to obtain more meaningful reconstructions of the WM pathways during glioma surgery.

## Data Availability Statement

The raw data supporting the conclusions of this article will be made available by the authors, without undue reservation.

## Ethics Statement

The studies involving human participants were reviewed and approved by Henan Provincial People's Hospital. The patients/participants provided their written informed consent to participate in this study.

## Author Contributions

ZS acquired and analyzed the data and wrote the manuscript. JY and YS acquired the data. XB and MW designed the research and acquired the data. CS analyzed the data and wrote the manuscript. JH supervised the research, designed the research, and wrote the manuscript. BN designed the research and wrote the manuscript. AZ and JA-C designed the research, acquired and analyzed the data, supervised the research, wrote the manuscript, and contributed the analytical tools. All authors contributed to the article and approved the submitted version.

## Conflict of Interest

The authors declare that the research was conducted in the absence of any commercial or financial relationships that could be construed as a potential conflict of interest.

## Publisher's Note

All claims expressed in this article are solely those of the authors and do not necessarily represent those of their affiliated organizations, or those of the publisher, the editors and the reviewers. Any product that may be evaluated in this article, or claim that may be made by its manufacturer, is not guaranteed or endorsed by the publisher.
